# The bovine mammosphere-derived epithelial cell secretome inhibits bacterial growth in vitro and contains peptidoglycan recognition protein 1

**DOI:** 10.1016/j.vas.2026.100643

**Published:** 2026-03-31

**Authors:** Rebecca M. Harman, Nikola Danev, Kelly A. Oxford, Leane Oliveira, Lucas Huntimer, Gerlinde R. Van de Walle

**Affiliations:** a Baker Institute for Animal Health, Cornell University, Ithaca, NY 14853, USA; b Elanco Animal Health, 450 Elanco Circle, Indianapolis, IN 46221, USA; c The Roslin Institute and Royal (Dick) School of Veterinary Studies, University of Edinburgh, Midlothian, Scotland, UK

**Keywords:** Bovine, Mastitis, Secretome therapy, Bacteria, Peptidoglycan recognition protein 1

## Abstract

Bovine mastitis, caused by bacterial infection of the mammary gland, leads to significant economic losses for the dairy industry because of decreased milk quality and yield. Mastitis is typically treated with conventional antibiotics (ABX) that clear bacteria, but do not address the milk loss associated with infection. Milk from cows being treated with ABX must be discarded leading to acute milk loss, and ABX do not heal milk-producing tissues that are damaged because of infection, leading to a permanent reduction in milk production when compared to pre-mastitis levels. The bovine mammosphere-derived epithelial cell (MDEC) secretome, consisting of all bioactive factors secreted by the cells, contains molecules that directly and indirectly promote tissue repair. This current study shows that the MDEC secretome also inhibits the growth of mastitis-causing bacteria *in vitro* in planktonic and biofilm assays. Secretome analysis by heat treatment, size fractionization, and extracellular vesicle isolation suggested that soluble proteins of different sizes, or in complexes, drive the anti-bacterial effects. We identified the antimicrobial protein peptidoglycan recognition protein 1 (PGLYRP1) in MDECs and detected it in the secretome. As knock down of PGLYRP1 expression in MDECs was unsuccessful using RNA interference and CRISPR-Cas9 gene editing, a direct link between the antimicrobial activity of the secretome and this protein could not be confirmed. The newly identified antimicrobial activity of the bovine MDEC secretome supplements its tissue reparative features, poising it as a potential adjunct or alternative therapy for bovine mastitis that may reduce acute milk loss and improve long term milk production.

## Highlights

• The bovine MDEC secretome possesses antimicrobial activity.
• The bovine MDEC secretome reduces the growth of mastitis pathogens *in vitro*.
• The bovine MDEC secretome contains the antimicrobial protein PGLYRP1.

## Graphical abstract

**Figure:**
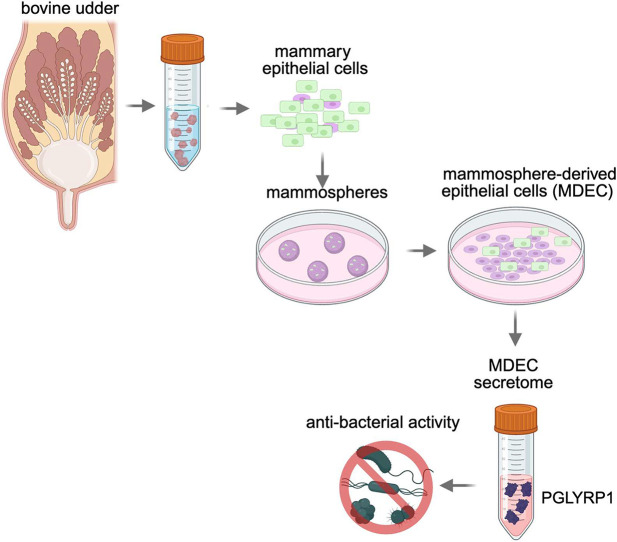

## 1 Introduction

Mastitis, inflammation of the mammary gland, is typically a result of bacterial infection (Cheng & Han, 2020). It is the most common infectious disease of dairy cattle and the single largest driver of economic loss for dairy farmers (Morales-Ubaldo et al., 2023). Antibiotics (ABX) are the gold standard for the treatment of bacterial mastitis, reducing bacteria and preventing disease progression (Cheng & Han, 2020; McDougall, 2002). Milk yields are negatively impacted by mastitis both acutely, because milk from cows treated with ABX must be discarded, and long term, due to udder damage caused directly by bacteria and indirectly by the host’s innate immune responses (Cheng & Han, 2020; St.Rose et al., 2003). ABX do not stimulate the regenerative processes necessary to repair the damage caused by the infection and cannot directly contribute to the recovery of pre-mastitis milk yields. Additionally, the ever-increasing threat of antimicrobial resistant (AMR) bacteria has led to significant efforts to reduce reliance on common ABX (Iwu et al., 2020). These limitations necessitate the exploration of adjunct or alternative treatments specific to the dairy industry, to address the need for a therapy with both antimicrobial and regenerative activities.

The field of regenerative medicine has increasingly focused on stem cells and biologically active molecules secreted by these cells, a.k.a the secretome, as potential therapeutics. The secretome can be defined as the total soluble and insoluble organic and inorganic molecules, lipids and extracellular vesicles (EVs; lipid bilayer-enclosed particles secreted by cells that mediate intercellular communication) released from cells under a defined time and under controlled conditions. (Da Silva et al., 2025). Previous work by our group identified the secretome of mammosphere-derived epithelial cells (MDECs) as a potential alternative to common mastitis treatments due to its regenerative and immunomodulatory properties. (Danev et al., 2024b, 2025; Harman et al., 2024; Ledet et al., 2018). We and others have shown that bovine epithelial cells also express antimicrobial proteins (Ledet et al., 2018; Swanson et al., 2004), a desirable feature for a mastitis treatment. MDECs are primary cell cultures derived from mammary tissues, which are enriched for epithelial stem and progenitor cells. First, single cells released from mammary tissue are briefly plated to separate mesenchymal and epithelial cells. Then, epithelial cells are cultured as mammospheres, a process that enriches for stem and progenitor cells (Ledet et al., 2018). Single cell RNA sequencing of bovine MDECs revealed that they are heterogeneous cell cultures that can be divided into 6 clusters based on the expression patterns of genes consistent with mammary epithelial cells as well as stem and progenitor cell markers and we speculated that these clusters represent multiple cell types, and cells at various stages of differentiation, that contribute to a broadly active secretome, responsible for its combinatorial regenerative, immunomodulatory and potential antimicrobial effects (Danev et al., 2025).

In this study, we report on the antimicrobial activity of the bovine MDEC secretome, collected as conditioned medium (CM), using both planktonic and biofilm assays. Biofilms are structured communities of bacteria encased in a self-created extracellular matrix. In mastitis, biofilms formed on udder tissues shield bacteria from the host immune system and administered antibiotics (Pederson et al., 2021), making them an important target for therapy. We confirm peptidoglycan recognition protein 1 (PGLYRP1) as a factor secreted by MDECs. PGLYRP1 is part of the peptidoglycan recognition protein (PGRP) family, a group of innate immunity proteins conserved across virtually all animals (Dziarski & Gupta, 2010). PGRP proteins are characterized by the ability to bind and disrupt peptidoglycan (PGN), an integral polymer in bacterial cell walls that maintains structure and function. Certain PGRPs, such as PGLYRP1, not only damage bacterial cell walls by interacting with PGN but also exert antimicrobial activity by interacting with other bacterial components (Tydell et al., 2006). Human, murine and bovine PGLYRP1 have been shown to be bactericidal for both gram-positive and gram-negative bacteria, as well as for certain fungal species (Osania et al., 2100; Tydell et al., 2002, 2006; Wang et al., 2003)*.* PGLYRP1 is found in the secretions of eosinophils (Dziarski & Gupta, 2010) and has been identified in both mammary and oral epithelial cells (Ledet et al., 2018; Uehara et al., 2005) as well as specifically in the lactating mammary gland (Kappeler et al., 2004).

The objectives of this study were to determine the antimicrobial activity of the bovine MDEC secretome and confirm PGLYRP1 as an active MDEC secreted factor. The antimicrobial nature, in addition to the immunomodulatory and regenerative features, of the MDEC secretome warrants further exploration of the potential of MDECs as an adjunct or alternative treatment for bovine mastitis.

## 2 Materials and methods

### 2.1 Cell culture and collection of conditioned medium (CM)

Bovine mammosphere-derived epithelial cells (MDECs) were isolated from lactating Holstein cows, as previously described (Ledet et al., 2018) and cultured in a sterile incubator at 37°C, 5% CO_2_ (standard conditions) in culture medium containing DMEM:F12 (Corning Inc, Corning, NY) at a 1:1 ratio supplemented with 10% fetal bovine serum (FBS, R&S Systems, Minneapolis, MN), 2% B27, 1% penicillin/streptomycin (P/S, both from Corning Inc), 10 ng/ml basic-fibroblast growth factor, and 10 ng/mL epidermal growth factor (both from Millipore Sigma, Burlington, MA). MDECs were characterized by the expression of proteins associated with mammary basal (cytokeratin 14), luminal (cytokeratin 18) and myoepithelial (alpha-smooth muscle actin) cells, as well as stem cells (CD29, CD4, CD49f), as published in an earlier study (Ledet et al., 2018). Bovine mammary fibroblasts were isolated as previously described (Ledet et al., 2018) and cultured under standard conditions in medium containing DMEM with 10% FBS and 1% P/S. Secreted factors were collected as CM by removing culture medium from flasks seeded with 13,333 cells/cm^2^ 24 h (h) earlier, rinsing the cell monolayer with phosphate buffered saline (PBS), and adding DMEM at 0.1 mL/cm^2^. After 24 h, CM was collected, centrifuged to remove cellular debris, and used in *in vitro* assays. To assess the effects of long-term storage of the bovine MDEC secretome, CM was frozen at -80°C for 3 months and thawed, or lyophilized under a refrigerated vacuum, held for 3 months, and then reconstituted to the original volume with sterile water before use in antibacterial assays.

### 2.2 Planktonic antibacterial assays

Antibacterial assays were carried out as previously described (Danev et al., 2023, 2025). Briefly, single clonal populations of mastitis pathogens *Staphylococcus (S.) aureus, Klebsiella (K.) pneumoniae, Escherichia (E.) coli* or*Methicillin-resistant Staphylococcus aureus (MRSA)* were inoculated into Luria Bertani (LB) broth (Thermo Scientific, Waltham, MA) and grown on a shaker overnight at 37°C. Bacteria were diluted to 100 colony forming units (CFU)/μl in LB broth, and 750 μl of CM was mixed with 250 μl of diluted bacteria. Inoculum was aliquoted into four 96-well plate wells at 200 μl/ well for a concentration of 5000 CFU of bacterial per well. DMEM, DMEM supplemented with 2% P/S (ABX) and fibroblast CM were included as positive and negative controls, respectively. For peptidoglycan recognition protein 1 (PGLYRP1) antibody blocking assays, CM was incubated with 3.0 µg/ml mouse anti-PGLYRP1 antibody (Cat # LS-C59, LSBio, Seattle, WA) or mouse IgG (Cat # ab18443, Abcam, Waltham, MA) for 1 h prior to mixing with bacteria and plating. To determine if PGLYRP1 inhibits bacterial growth in our system, recombinant bovine PGLYRP1 (Cat # MBS1344010 MyBiosource, San Diego, CA) was diluted in DMEM to 5, 10, 50 and 100 µg/ml, and 750 μl of diluted PGLYRP1 was mixed with 250 μl of diluted bacteria and plated as described above. For all bacterial assays, plates were incubated for 24 h at 37°C and absorbance was measured at 600 nm every 30 min (min) on a Tecan Infinite 200 Pro plate reader (Tecan, Morrisville NC).

### 2.3 Biofilm assays

To determine the antibacterial effect of MDEC CM on *MRSA* grown in biofilms, the microtiter dish biofilm assay was used, as described previously (Marx et al., 2020). *MRSA* cultures in LB broth were diluted 1:2 during the exponential growth phase, and 50 µl were plated in triplicate wells of 96 well u-bottom plates. An equal volume of MDEC CM or the controls DMEM, fibroblast CM and ABX, was added to each well with biofilms for 24 h at 37°C. After 24 h, an additional 50 µl of MDEC CM or control medium were added and plates were returned to 37°C for another 24 h. Medium was removed from wells and biofilms were gently rinsed with PBS and stained with 0.1% crystal violet (Thermo Scientific) diluted in 20% ethanol for 5 min. After staining, wells were washed twice with distilled water and stained biofilms were air dried for at least 2 h. Crystal violet was solubilized in 125 µl 30% glacial acetic acid, transferred to flat bottom plates and absorbance was measured at 550 nm on the Infinite 200 Pro plate reader.

Viability of *MRSA* in biofilms was determined by plating bacteria and treatments at a 1:1 ratio, for a total volume of 400 µl, in 24 well plates that were fitted with glass coverslips. Plates were incubated and treated with 200 µl volume according to the schedule described above. After 48 h, cells were stained using live/dead cell staining reagents (Abcam) and imaged using a confocal laser scanning microscope (Olympus, Center Valley, PA). Live cells in images (10 fields per sample) were quantified with Fiji ImageJ software (https://imagej.ned/Fiji).

### 2.4 Heat-treatment and size fractionation of conditioned medium (CM) and isolation of extracellular vesicles (EVs)

For heat-treatment, CM samples were placed in a water bath that was maintained at 95°C for 10 min, after which they were rapidly cooled back on ice to room temperature. To separate the components of CM by size, 6 ml of freshly collected CM was dispensed into Amicon Ultra 15 Ultracel 30 K centrifugal filters (Merck, Darmstadt, Germany). Centrifugal filters were spun, as directed by manufacturer, at 5000 x g for 40 min at room temperature in a fixed-angle rotor. Flowthrough was brought to 6 ml with DMEM and used as <30 kDa CM, and concentrated solute was brought to 6 ml with DMEM and used as >30 kDa CM. Extracellular vesicles were isolated by ultracentrifuging CM at 30,000 x g for 2 h with brake set to slow at 4°C in an Optima L-90 K ultracentrifuge (Beckman Coulter, Brea, CA). Supernatant was transferred to new tubes and used as EV-free CM. Pellets were resuspended in 6 ml DMEM and used as the EV-containing fraction.

### 2.5 Quantitative reverse-transcriptase PCR (qRT-PCR)

qRT-PCR was done exactly as described previously (Danev, Li et al., 2024a). Briefly, RNA was extracted from cells with a RNeasy Mini Plus kit (Qiagen, Germantown, MD) following manufacturer’s instructions. An iScript gDNA Clear cDNA synthesis kit (Bio-Rad, Hercules, CA) was used to convert RNA to cDNA, which was amplified using PowerTrack SYBR Green MasterMix (Applied Biosystems, Waltham, MA). qPCR was performed in QuantStudio 3 thermal cycler (Applied Biosystems). Primer sequences were as follows: peptidoglycan recognition protein 1 (*PGLYRP1*) forward (5′−3′) TCCAGCCCCGGCCCTCATAC, reverse (5′−3′) ACTGCGGCAGCATCGTGTCC, Glyceraldehyde 3-phosphate dehydrogenase (*GAPDH*) forward (5′−3′) CTCCCAACGTGTCTGTTGTG, reverse (5′−3′) CCTGCTTCACCACCTTCTTG. Data were analyzed in Microsoft Excel using the -ΔΔCt method and visualized in Prism 10 (GraphPad, Boston, MA).

### 2.6 Immunofluorescent (IF) labeling, imaging, and quantification

IF imaging was performed exactly as previously described (Danev, Li et al., 2024a). Cells grown for 24 h on glass coverslips were fixed with 4% paraformaldehyde (Thermo Scientific) for 10 min and permeabilized with 0.1% Triton (MilliporeSigma) for 10 min. Permeabilized cells were blocked in PBS supplemented with 10% bovine serum albumin (BSA; Thermo Scientific). Cells were then incubated with polyclonal rabbit anti-human PGLYRP1 antibodies (Cat# SAB1411594, MilliporeSigma), diluted 1:100 in PBS, overnight at 4°C, washed three times with PBS, and incubated with polyclonal DyLight650-conjugated goat anti-rabbit IgG, diluted 1:500 in PBS (Cat# SAS-10034,Thermo Scientific) for 1 h at room temperature. After washing three times with PBS, 4′,6-diamidino-2-phenylindole (DAPI) (Thermo Scientific) diluted at 1:20,000 in PBS was added for 5 min at room temperature, and cells were washed with PBS. Coverslips were attached to glass slides using aqueous mounting medium (Dako, Santa Clara, CA) and visualized with a confocal laser scanning microscope (Zeiss, Oberkochen, Germany). Quantification of IF images was performed in FIJI (Schindelin et al., 2012) by measuring mean fluorescent intensity per field and dividing by the number of nuclei in the field (to determine MFI per cell), in 10 fields per condition per experiment. Data were plotted and visualized in Prism 10.

### 2.7 Knockdown of bovine peptidoglycan recognition protein 1 (PGLYRP1)

RNA interference (RNAi) using short interfering RNA (siRNA) was performed exactly as described previously (Harman et al., 2024). Briefly, Silencer Select siRNAs targeting bovine *PGLYRP1* were designed (Thermo Scientific). Silencer Select Negative Control #2 was used as a non-specific (scramble) control, after it was aligned to the bovine genome (ARS-UCD2.0) and determined to be non-specific to any coding or non-coding region. MDECs were counted manually using a hemacytometer and approximately 1  × 10^4^ MDECs were seeded per cm^2^. Lipofectamine RNAiMAX Reagent (Thermo Scientific) /siRNA complexes were generated, incubated for 5 min, and used to transfect cell cultures for 24 h with 5 nM siRNA, after which the medium was changed to culture medium, and cells were used for downstream assays.

CRISPR-Cas9 gene editing with TrueCut Cas9 Protein v2 (Thermo Scientific) was used to delete *PGLYRP1* from bovine MDECs as per manufacturer’s instructions. TrueCut Cas9 protein *PGLYRP1* 1 of 3 specific guide RNAs (gRNAs) were mixed to form complexes. Multiple gRNAs were used to reduce off target activity. Complexes of Cas9 and each gRNA were added to MDECs in suspension culture before electroporation using a Neon NxT system (Thermo Scientific). After 3 days in culture, a portion of transfected MDECs was collected for verification of transfection efficiency. To this end, cell cultures were expanded and stained with 2 µg/ml propidium iodine (PI). Stained cells were sorted using a BD FACS Melody cell sorter (BD, Franklin Lakes, NJ). One thousand live cells (PI negative) were plated at 1 cell per well in 96-well culture plates for clonal expansion and verification of *PGLYRP1* deletion.

### 2.8 Western blots

Western blotting was performed with minor modifications from previously published protocols (Ledet et al., 2018) Briefly, after 1 × 10^6^ cells were pelleted and lysed or 1 ml of CM was collected, samples were incubated with Halt protease inhibitor (Thermo Scientific). Protein concentration was determined using a bicinchoninic acid (BCA) protein assay (Thermo Scientific), and lysates/CM were diluted for equal loading. Reducing sample buffer was added and samples were heated for 45 s (*sec*) at 60 °C and immediately cooled to RT. Samples were run on a sodium dodecyl sulfate polyacrylamide gel for electrophoresis, then transferred onto polyvinylidene difluoride membranes (Bio-Rad). Membranes were blocked in 5% BSA diluted in Tris-buffered saline (TBS) (blocking buffer) for 1 h at room temperature, and then washed three times with TBS. Membranes were incubated sharking, overnight in monoclonal anti-mouse peptidoglycan recognition protein 1 (PGLYRP1) antibodies (LS Bio, Lynwood, WA, Cat# LS-C579), diluted 1:200 in blocking buffer, at 4°C. After 3 washes in TBS, for 2 min each, membranes were incubated with polyclonal HRP-conjugated goat anti-mouse IgG antibodies (Jackson ImmunoResearch, West Grove, PA, Cat# 115-035-062), diluted at 1:20,000 in blocking buffer, for 1 h at room temperature. Blots were washed in TBS three times and visualized by Clarity Western ECL (BioRad), as per manufacturer’s protocols.

### 2.9 Statistical analysis

Unpaired t tests with Welch’s corrections were used to compare two groups. One-way ANOVA followed by Tukey’s multiple comparisons test were used to compare data from >2 groups. Analysis was done using Prism software (GraphPad, Boston, MA). Experiments were each performed with CM generated from MDECs isolated from three individual cows. Data shown are the mean of 3 experiments, error bars indicate standard deviations. Differences with a p-value < 0.05 are considered significant.

## 3 Results and discussion

**Fig. 1:**
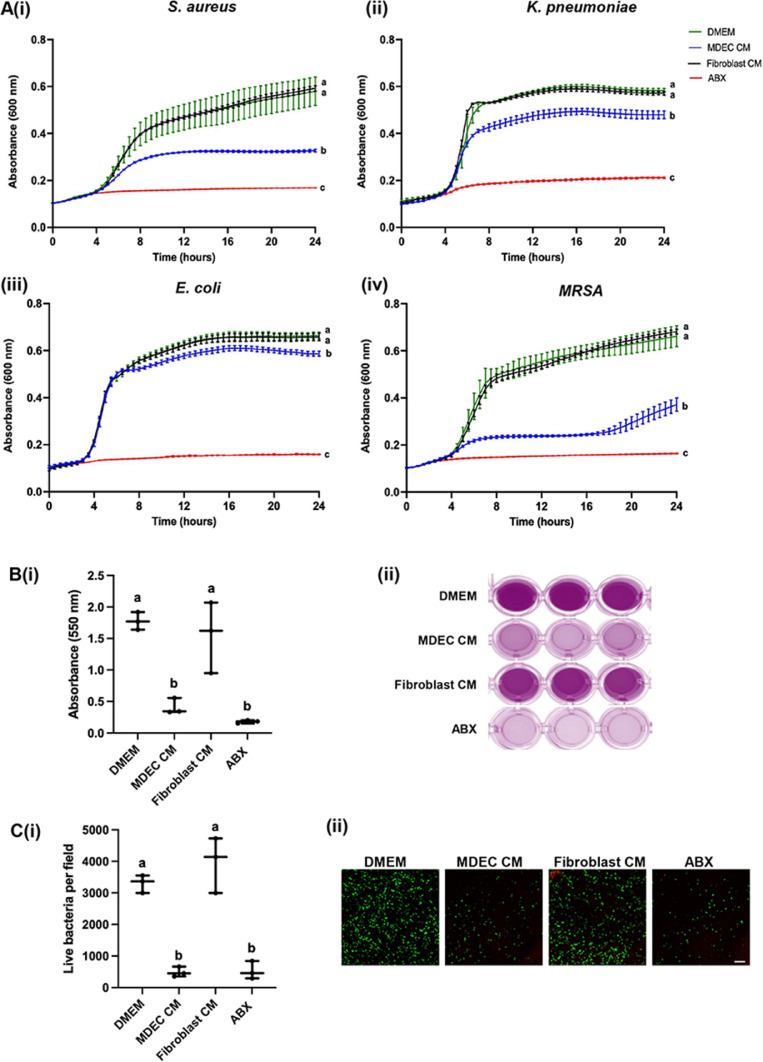
Bovine mammosphere-derived epithelial cell (MDEC) conditioned medium (CM) inhibits bacteria in planktonic cultures and biofilms. (A)(i-iv). Planktonic growth curves of 4 common mastitis-causing pathogens when exposed to culture medium (DMEM), MDEC CM, fibroblast CM or antibiotics (ABX) for 24 h (h) measured by absorbance at 600 nm. **(B)(i**). Crystal violet incorporation in *Methicillin-resistant Staphylococcus aureus (MRSA)* biofilms measured at an absorbance 0f 550 nm. **(ii).** Representative images of crystal violet stained biofilms.**(C)(i).** Numbers of live cells in *MRSA* biofilms labeled with fluorescent live (green) and dead (red) dyes formed in DMEM, MDEC CM, fibroblast CM or ABX for 48 h. **(ii).** Representative images of stained cells in biofilms. Scale bar = 20 µm. *n* = 3. Data on graphs indicate means ± standard deviations. Different letters on graphs indicate significant differences. *p* < 0.05.

Planktonic assays were used to determine if mammosphere-derived epithelial cell conditioned medium (MDEC CM) has anti-bacterial properties. MDEC CM decreased the density of all tested mastitis-causing bacteria as follows: *Staphylococcus (S.) aureus* by 37.18%,*Klebsiella (K.) pneumoniae* by 15.70%,*Escherichia (E.) coli* by 21.05%, and*Methicillin-resistant Staphylococcus aureus (MRSA)* by 47.40%, as compared to DMEM control (Fig. 1A(i-iv)). Mammary fibroblast (fibroblast) CM was included as a cellular control and DMEM with antibiotics (ABX) was included as a positive control. These data are based on absorbance readings of planktonic bacterial cultures in 96-well plate wells, with higher absorbance readings indicating a higher density of bacteria per well. Future assays with a broader range of bacteria, for example multiple different strains of each species, are warranted to address the observation that MDEC CM affects the density of different mastitis-causing bacteria to different degrees and at seemingly different times in the planktonic assays.

As biofilms play a role in the persistence of mastitis infections and the resistance of bacteria to treatment, we applied MDEC CM to an *in vitro* model of *MRSA* biofilm formation, in which biofilm mass is determined by crystal violet incorporation, with higher absorbance readings indicating more bacteria per well (O’Toole, 2011). MDEC CM, but not fibroblast CM, significantly reduced biofilm mass by 76.76%, as compared to DMEM control (Fig. 1B(i)). As expected, the positive control consisting of a high dose of ABX significantly reduced biofilm mass (Fig. 1B(i)). Representative images of biofilms stained with crystal violet are shown in Fig. 1B(ii). The effect of MDEC CM on biofilms was also determined by the visualization and quantification of live bacteria using confocal microscopy. Compared to the DMEM control, 85.06% fewer live cells were detected in biofilms treated with MDEC CM. There was no difference in live cell number in biofilms treated fibroblast CM compared to the DMEM control, and treatment with ABX significantly reduced live cells (Fig. 1C(i)). Representative images of live cells in biofilms are included in Fig. 1C(ii)). We concluded that MDEC CM can reduce *MRSA* accumulation in biofilms as well as in planktonic culture.

**Fig. 2:**
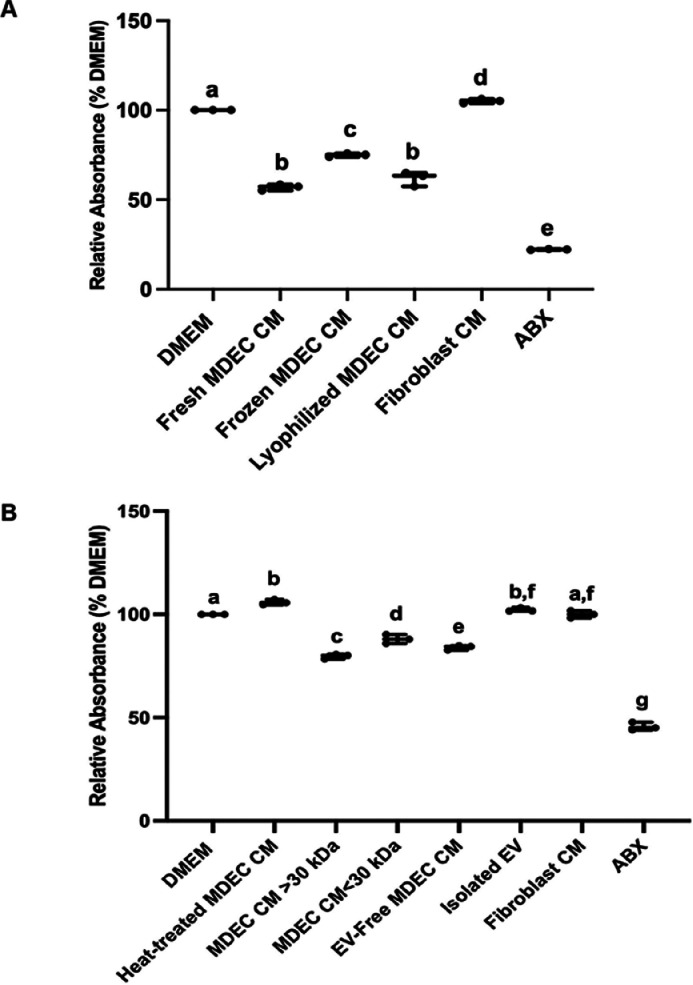
The inhibition of bacterial growth by bovine mammosphere-derived epithelial cell (MDEC) conditioned medium (CM) is likely due to protein activity. **(A).** Relative absorbance values of planktonic *Methicillin-resistant Staphylococcus aureus (MRSA)* cultures after 24 h (h) in culture medium (DMEM), MDEC CM, frozen/thawed MDEC CM, lyophilized/reconstituted MDEC CM, fibroblast CM or antibiotics (ABX). **(B).** Relative absorbance values of planktonic *MRSA* after 24 h when cultured in DMEM, heat-treated MDEC CM, MDEC CM fraction larger than 30 kDa, MDEC CM fraction smaller than 30 kDa, extracellular vesicle (EV)-free MDEC CM, isolated EV, fibroblast CM or ABX. *n* = 3 Data on graphs indicate means ± standard deviations. Different letters on graphs indicate significant differences. *p* < 0.05.

For practical clinical use, mastitis therapies must be stored long term without losing effectiveness. We tested the effects of MDEC CM that was either frozen or lyophilized for 3 months on *MRSA* growth in planktonic assays, as proof-of-concept. Compared to the DMEM control, fresh, frozen/thawed and lyophilized/reconstituted MDEC CM each reduced the density of *MRSA* at 24 h (h), and the fibroblast CM and ABX controls both exhibited the expected effects (Fig. 2A). Lyophilized/reconstituted CM reduced the density of *MRSA* to the same degree fresh MDEC CM did, while frozen/thawed CM was slightly less effective (Fig. 2A). This suggests that lyophilization may be a more appropriate means to store MDEC CM long term than freezing.

Certain proteins and lipids have been shown to exhibit antimicrobial effects against various bacterial pathogens, including *MRSA* (Le et al., 2022). To assess whether proteins and/or lipids mediate the antimicrobial properties of the MDEC secretome, CM was heated to 95°C for 5 min. This temperature is sufficient to denature and deactivate most proteins, but the function of lipids should not be impacted as the simple ketoacyl and isoprene chain structures of lipids cannot be heat-denatured (Fahy et al., 2011). After heat treatment, MDEC CM did not decrease the absorbance of values of wells containing *MRSA* as compared to the DMEM control (Fig. 2B), suggesting that proteins most likely contribute to the antimicrobial properties of bovine MDEC CM. Proteins vary in mass, from 1 kDa to over 3000 kDa. To determine whether smaller proteins, such as antimicrobial peptides (AMPs) that are often <10 kDa, or larger proteins/protein complexes play a role in the antimicrobial effects of the MDEC secretome, fractionation of CM using a cutoff of 30 kDa was performed. Both fractions reduced the density of *MRSA* when compared the DMEM control (Fig. 2B). These results suggest a potential synergistic antimicrobial effect between several proteins, potentially a smaller and larger protein, or a protein complex. Combining different antimicrobial peptides to achieve enhanced antibacterial effects is an approach used by researchers to create effective therapies to reduce the development of antibiotic resistance (Gagandeep et al., 2024). Dissecting the MDEC secretome, which may rely on several proteins to exert antimicrobial effects, could provide information to inform this approach to therapeutic development.

Extracellular vesicles are lipid-bilayer delimited particles that are larger than 30 kDa, and act as cellular messengers by delivering bioactive proteins, lipids and nucleic acids to target cells. EVs have been shown to have antimicrobial effects (Capriotti et al., 2022; Zhao et al., 2022) so we isolated EVs from MDEC CM, to compare the antibacterial role of EVs to that of soluble molecules. EVs were separated from the soluble fraction of MDEC CM using an ultracentrifugation-based protocol, after which each fraction was diluted back to the original volume. EV-free CM, but not EVs, significantly reduced the density of planktonic *MRSA* at 24 h (Fig. 2B), suggesting soluble proteins are responsible for the antimicrobial activity of MDEC CM. Fibroblast CM and ABX controls both had the anticipated effects on *MRSA* density (Fig. 2B). Collectively, these findings indicate that the MDEC secretome contains soluble proteins of different sizes, or in complexes, that drive the anti-bacterial effects.

Previously published MDEC single cell RNA sequencing (scRNA-seq) data (Danev et al., 2025) and MDEC CM mass spectrometric data (Ledet et al., 2018) were mined for transcripts and proteins with known antimicrobial properties, to identify the proteins most likely involved in the antimicrobial activity of MDEC CM (Table 1). Bovine peptidoglycan recognition protein 1 (PGLYRP1), was confirmed in both transcriptomic and proteomic analyses and met the criteria of mass, as it is <30 kDa and oligomerizes into functional complexes larger than 30 kDa (Tydell et al., 2002, 2006). PGLYRP1 is a highly conserved antimicrobial protein of approximately 19 kDa that forms multimeric complexes and has been reported in insects, rodents, cattle, camels, pigs and humans. In mammals, it has been isolated from bone marrow, lymph nodes and mammary gland tissue (Kappeler et al., 2004; Tydell et al., 2002, 2006; Yashin et al., 2021). Moreover, bovine PGLYRP1 has been shown to be strongly bactericidal to multiple pathogens, including *S. aureus*, the non-antimicrobial resistant form of *MRSA* (Tydell et al., 2002, 2006)*.*

**Table 1:** Antimicrobial transcripts/proteins identified in single cell RNA-seq analysis of mammosphere-derived epithelial cells (MDECs) and mass spectrometric analysis of MDEC conditioned medium (CM).

Transcript/Protein	scRNA-seq	Mass spectrometry (Molecular weight in kDa)
Cathelicidin-7		X (18)
Cathepsin	X	X (52)
Contactin-1		X (110–120)
Lactoferrin	X	X (76–80)
Lactoperoxidase	X	
Mannose-binding protein C		X (25)
Pantetheinase	X	X (57)
Peptidoglycan recognition protein 1	**X**	**X (19)**
Protein S100-A11	X	X (17)

**Fig. 3:**
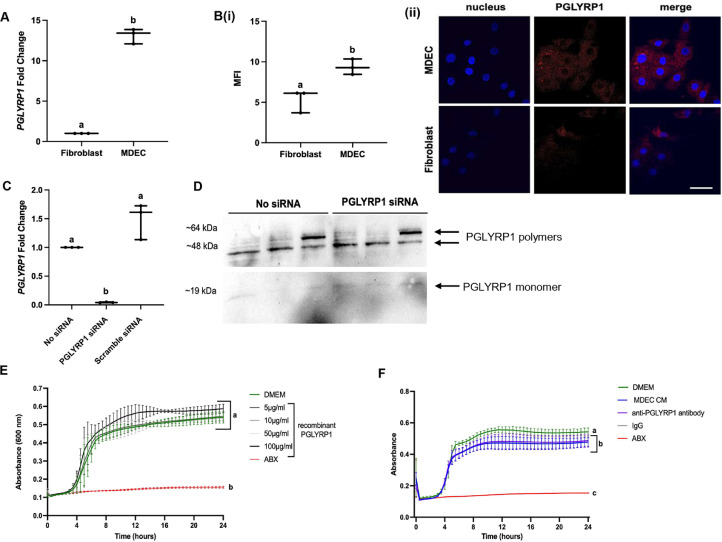
Peptidoglycan Recognition Protein 1 (PGLYRP1) is abundant in bovine mammosphere-derived epithelial cells (MDECs) but is not clearly involved in the antimicrobial action of MDEC conditioned medium (CM). **(A).***PGLYRP1* transcript expression in bovine fibroblasts and MDECs as determined by qRT-PCR. **(B)(i).** Fluorescent PGLYPR1 antibody binding on bovine fibroblasts and MDECs to detect protein expression, expressed as mean fluorescence intensity (MFI) **(ii).** Representative images. Scale bar = 50 µm. **(C).***PGLYRP1* transcript expression in MDECs after RNA interference (RNAi) using short interfering RNA (siRNA) specific to *PGLYRP1* as determined by qRT-PCR. **(D).** Western blot analysis of PGLYRP1 protein expression in MDECs after RNA interference (RNAi) using short interfering RNA (siRNA) specific to *PGLYRP1.* Arrows point to PGLYRP1 in monomeric and polymeric forms. **(E).** Planktonic growth curves of *Methicillin-resistant Staphylococcus aureus (MRSA)* when exposed to culture medium (DMEM), DMEM containing recombinant bovine PGLYRP1 at various concentrations, or antibiotics (ABX) for 24 h (h) measured by absorbance at 600 nm. **(F).** Planktonic growth curves of *MRSA* when exposed to DMEM, MDEC CM, MDEC CM pre-treated with an anti-PGLYPR1 antibody, MDEC CM pre-treated with rabbit IgG, or ABX for 24 h measured by absorbance at 600 nm. *n* = 3. Data on graphs indicate means ± standard deviations. Different letters on graphs indicate significant differences. *p* < 0.05.

To assess whether PGLYRP1 mediates the antimicrobial effects of MDEC CM, we first compared gene expression levels of *PGLYRP1* between MDECs and fibroblasts, as fibroblast CM had no or minimal antimicrobial effects (Fig. 1A,(i-iv) B&C). MDECs showed a significantly higher gene expression of *PGLYRP1*, as determined by qRT-PCR, compared to fibroblasts (Fig. 3A). Also, the mean fluorescent intensity (MFI) of the fluorophore bound anti-PGLYRP1 antibody was significantly higher in labeled MDECs than in fibroblasts (Fig. 3B(i)), suggesting that PGLYRP1 protein is also more abundantly expressed by MDECs. Representative images of PGLYRP1 expression are shown in Fig. 3B(ii).

We then proceeded to use standard techniques to knock down PGLYRP1 expression in MDECs, in order to generate PGLYRP1-free CM for use in planktonic bacterial assays. If CM from MDECs unable to express PGLYRP1 no longer reduce bacterial growth, this would indicate that PGLYRP-1 is required for the antibacterial effects. We started with RNA interference (RNAi) using short interfering RNA (siRNA) designed to cleave *PGLYRP1* mRNA, targeting it for degradation. RNAi with *PGLYRP1* siRNA did decrease *PGLYRP1* transcript expression in MDECs, while RNAi with a non-specific “scramble” siRNA did not (Fig. 3C). However, there was no observable decrease in PGLYRP1 protein after RNAi as assessed by Western Blot (Fig. 3D), suggesting that siRNA is not a reliable means to knock down PGLYRP1 protein in MDECs. Although mRNA abundance can be a strong indicator of protein abundance, multiple factors may contribute to discrepancies between the two. For example, large quantities of mRNA may be transcribed but only a fraction of them may be translated. Alternatively, amplification systems exist that elevate protein translation from small quantities of mRNA. These compensatory mechanisms may explain the unchanged PGLYRP1 protein levels, despite successful RNAi. Moreover, it has also been shown that the half-life of the targeted protein plays a significant role in the effectiveness of RNAi in decreasing protein abundance. Protein turnover is a key regulator of protein homeostasis, yet an increasing number of proteins have been identified as being able to evade normal protein degradation. While half-life and protein degradation evasion assays have not been conducted directly on PGLYRP1, it has been found that this protein can persist in the cellular membrane, where it can embed itself for longer periods of time (Kashyap et al., 2011). This prolonged persistence could account for the unsuccessful attempts to ablate PGLYRP1 using RNAi.

We next attempted to interrupt *PGLYRP1* gene expression using CRISPR-Cas9 editing. Confirmational genetic tests showed that *PGLYRP1* was deleted in about 30% of the cells in the targeted MDEC culture (data not shown). From that culture, 1000 propidium-iodide (PI) negative (live) MDECs were sorted and distributed at a frequency of one cell per well, to generate clonal populations. From previous work in our lab, we expected about 10% of a bulk bovine MDEC culture could grow as clones (Miller et al., 2022), and of those, 30% would not express *PGLYRP1.* Unfortunately, after transfection with CRISPR-Cas9 reagents, MDECs did not proliferate under clonal conditions and populations derived from single cells were not generated, indicating that this method of gene editing is not appropriate for knocking down gene expression in MDECs.

Since we did not successfully knock down *PGLYRP1* in MDECs, and could not produce CM lacking this protein, we took indirect approaches to determine whether PGLYRP1 in bovine MDEC CM could be playing a role in reducing bacteria in planktonic cultures. We first performed a planktonic anti-*MRSA* assay using recombinant bovine PGLYRP1 at four concentrations (5, 10, 50 and 100 µg/ml) based on earlier work by another group (Liu et al., 2000) to see if the recombinant protein alone could reduce *MRSA.* No reduction of *MRSA* was observed in the planktonic assay, at any of the recombinant PGLYRP1 concentrations tested (Fig. 3E). The seemingly contradictory results of our study and the previously published data could be because we attempted to inhibit the growth of a different *Staphylococcus s*pecies. We also used recombinant bovine PGLYRP1 to maintain species consistency in our system, rather than the recombinant mouse PGLYRP1 that was the focus of the earlier work (Liu et al., 2000), for wich we do not know if this recombinant version of the protein is in its active form. Native bovine PGLYRP1 does not require post-translational phosphorylation to be active, but its antibacterial activity is primarily linked to its ability to form disulfide-linked homodimers (Tydell et al., 2002, 2006). Another potential explanation could be based on the genetics of our model system. Single nucleotide polymorphisms (SNPs) in the *PGLYRP1* gene have been associated with the incidence of bacterial disease in cattle, including mastitis resistance (Pant et al., 2011; Wang et al., 2013). However, since we did not observe varying effects of the MDEC CM against *MRSA* based on MDEC cell lines used (CM from all tested cell lines inhibited *MRSA* growth) it seems unlikely that the antimicrobial effects of MDEC CM are a result of *PGLYRP1* SNPs in bovine MDECs.

We then incubated MDEC CM with an anti-PGLYRP1 antibody, at a concentration we previously used to block antimicrobial peptide activity with other antibodies in equine stem cell CM (Harman et al., 2017), prior to using it in an anti-*MRSA* assay. No change in antibacterial activity was observed when CM was pre-treated with antibody, indicating that the anti-PGLYRP1 antibody did not block the active molecules in the CM (Fig. 3F). The negative result of this experiment does not exclude the activity of PGLRYP1 as an inhibitor of *MRSA* growth in our assays. Kinetics of antibody blocking are difficult to optimize, and although we did detect PGLRYP1 in Western blots using this antibody, we do not know if this antibody binds to and/or functionally blocks the region of PGLYRP1 that is responsible for bacterial growth reduction.

This study showed that bovine MDECs secrete factors that decrease the density of mastitis-associated bacteria in planktonic culture and in biofilms and decrease the number of live bacteria in biofilms. In addition, we found that MDEC CM retains its antimicrobial activity after storage in frozen or lyophilized forms. Heat-treatment, size separation and isolation of EV suggested that the antimicrobial factors are soluble proteins of different sizes, or in complexes. Global RNA and protein analysis, confirmed by RT-PCR and Western Blotting respectively, indicated that bovine MDEC CM contains the antibacterial protein PGLYRP1, which we focused on as the most likely active bioactive factor based on physical qualities. As we could not confirm in the present study that PGLYPR1 is responsible for the antimicrobial activity of MDEC CM, future work will focus on evaluating additional proteins/protein complexes present in the CM that can contribute to this effect. The antibacterial nature, as well as the previously established tissue reparative qualities of MDEC CM, justify the exploration of this biologic as a therapy for bovine mastitis that may reduce milk waste and improve milk production, thus, reducing the negative impact of mastitis on the dairy industry.

## Funding

This work was in part supported by the National Institute of Food and Agriculture, U.S. Department of Agriculture through a Hatch Grant # NYC-473426 and a grant from the Foundation for Food and Agricultural Research (FFAR) # CA20-SS-0000000004, which includes matching funds from Elanco Animal Health Incorporated and New York Farm Viability Institute (NYFVI), to GVdW.

## Ethical statement

Statement of co-authors: The manuscript was reviewed by all authors who approve of its submission to *Veterinary and Animal Science*.

Statement of publication material: The material submitted for publication has not been previously reported and is not under consideration for publication elsewhere.

## CRediT authorship contribution statement

**Rebecca M. Harman:** Writing – original draft, Visualization, Validation, Supervision, Methodology, Formal analysis. **Nikola Danev:** Writing – review & editing, Visualization, Validation, Methodology, Formal analysis. **Kelly A. Oxford:** Writing – review & editing, Validation, Investigation. **Leane Oliveira:** Writing – review & editing, Resources, Conceptualization. **Lucas Huntimer:** Writing – review & editing, Resources, Conceptualization. **Gerlinde R. Van de Walle:** Writing – review & editing, Supervision, Project administration, Funding acquisition, Conceptualization.

## Declaration of competing interest

The authors declare that they have no known competing financial interests or personal relationships that could have appeared to influence the work reported in this paper.
